# Neutral- and Multi-Colored Semitransparent Perovskite Solar Cells

**DOI:** 10.3390/molecules21040475

**Published:** 2016-04-11

**Authors:** Kyu-Tae Lee, L. Jay Guo, Hui Joon Park

**Affiliations:** 1 Department of Electrical Engineering and Computer Science, The University of Michigan, Ann Arbor, MI 48109, USA; ktlee@umich.edu; 2 Division of Energy Systems Research, Ajou University, Suwon 16499, Korea; 3 Department of Electrical and Computer Engineering, Ajou University, Suwon 16499, Korea

**Keywords:** perovskite, solar cells, semitransparent

## Abstract

In this review, we summarize recent works on perovskite solar cells with neutral- and multi-colored semitransparency for building-integrated photovoltaics and tandem solar cells. The perovskite solar cells exploiting microstructured arrays of perovskite “islands” and transparent electrodes—the latter of which include thin metallic films, metal nanowires, carbon nanotubes, graphenes, and transparent conductive oxides for achieving optical transparency—are investigated. Moreover, the perovskite solar cells with distinctive color generation, which are enabled by engineering the band gap of the perovskite light-harvesting semiconductors with chemical management and integrating with photonic nanostructures, including microcavity, are discussed. We conclude by providing future research directions toward further performance improvements of the semitransparent perovskite solar cells.

## 1. Introduction

Increasing effort is being applied to develop multi-functional solar cells that integrate new capabilities for creating various or neutral colors in a desired manner, thereby opening up the possibility of numerous applications, including building-integrated photovoltaic (BIPV) systems, photon energy conversions, tandem cells, wearable electronics, and energy-saving colored display technologies [1,2,3,4,5,6,7,8,9,10,11,12,13,14,15,16,17,18,19]. Although their power conversion efficiency (PCE) is relatively lower than conventional opaque solar cells due to the transmitted light through devices, which cannot be utilized for solar power generation, they can be integrated harmoniously into any type and surface of buildings, thus enabling a large portion of the entire building envelope, such as façade, skylights, walls, and windows to be employed for electricity generation from solar energy. There have been a large number of attempts to give additional aesthetic functionalities to the existing solar cells. Dye-sensitized solar cells (DSSC) have shown great promise in creating semitransparent solar cell devices with generating vivid colors by a proper mixture of organic dyes. However, different organic dye materials should be used to tune the semitransparent colors, and more importantly, it is hard to observe clear images through the DSSC devices, which is attributed to the scattering arising from a mesoporous structure in an intrinsic device configuration of the DSSC [20,21]. For easy color-tunable properties, plasmonic color filtering schemes have been integrated with organic solar cells, where subwavelength metallic gratings with different periodicity were utilized to excite surface plasmon resonances (SPR) at visible frequencies, and hence produced the desired colors [22]. Both extremely short exciton diffusion length and large energy losses occurring during charge transfer processes at donor/acceptor interfaces of the photoactive layer in the organic solar cell, however, resulted in the limited PCE. To address these challenges, various structural color filters based on optical micro- and nano-cavities have also been incorporated with the industrially-mature and proven amorphous silicon (a-Si) solar cell technologies [1,18]. However, due to their intrinsic property associated with irregular atomic arrangements that lead to a non-crystalline structure, unusual electrical behaviors are often caused by a dangling bond in the amorphous structure serving as defects in the disordered atomic network. Although the density of the dangling bonds can be significantly reduced by the passivation of the a-Si material with hydrogen, thus forming hydrogenated a-Si (a-Si:H) that exhibits improved electronic properties, the performance degradation when exposed to sunlight, known as the Staebler–Wronski effect, is the biggest challenge still to be addressed [23]. Therefore, there is a strong demand for a new solar cell material platform that offers performance improvements with simple processibility at low temperatures and low costs.

Organometal trihalide perovskite semiconductors have emerged as highly attractive light-absorbing materials for solar cell applications due to their ability to be easily processed with high efficiency and low cost over a large area. Moreover, these materials have intriguing characteristics, such as direct band gap, long carrier diffusion length, and high carrier mobility [24,25,26]. An enormous amount of effort has been recently devoted to improving the performance of the perovskite solar cells, achieving over 20% of the PCE that approaches the efficiency of commercial silicon (Si) solar cells while preserving the applicability to low-cost solution processing at low temperatures [27,28,29]. Considerable research has been conducted in the area of improving the crystallization of the perovskite semiconducting materials, optimizing the processing conditions, exploiting new device architectures, finding efficient interfacial layers, and engineering the composition of the perovskite semiconductors for further enhancing the PCE of the perovskite solar cells. Furthermore, to commercialize this technology, additional issues such as the toxicity of lead (Pb, usually a component of organometal trihalide perovskite [30]), the high-sensitivity of perovskite crystal toward the humidity from ambient water [31], and the relatively high cost of organic buffer layers like 2,2’,7,7’-tetrakis(*N*,*N*-di-*p*-methoxyphenylamine)-9,9-spirobifluorene (Spiro-OMeTAD) [32] should be solved. At the same time, a significant emphasis has also been placed on developing semitransparent and colored perovskite solar cells by controlling morphology, integrating with photonic nanostructures or transparent electrodes, and engineering the band gap of the perovskite absorbers. In particular, tremendous progress has been made in exploring various kinds of transparent electrodes, such as thin metal films sandwiched or covered by wide band gap dielectric materials, metallic nanowires, graphenes, carbon nanotubes, transparent conductive oxides, and conducting polymers [3,7,10,13,14,33,34,35,36,37,38,39,40,41,42,43,44,45,46,47,48,49,50,51,52].

Here we provide a review that focuses on the recent developments in semitransparent perovskite solar cells creating neutral- and multi-colors. We first summarize a wide variety of approaches, which includes thin metal films, low-temperature solution-deposited metallic nanowires, transparent conductive oxides, carbon nanotube- and graphene-based electrodes, and morphological controls, to achieve the neutral-colored semitransparent perovskite solar cells. In addition, colored perovskite solar cells that exploit the microcavity and band gap engineering are examined. Current issues and guidance for future perspectives to further improve the performance characteristics of the semitransparent solar cells are discussed at the end.

## 2. Neutral-Colored Semitransparent Perovskite Solar Cells

### 2.1. Thin Metal Electrodes

A significant number of studies on semitransparent perovskite solar cells have been dedicated to developing transparent electrodes, engineering the band gap of the perovskite semiconductors, and architecting the new device structures. In particular, planar device structure configurations have been widely adopted due to their simplicity and ability to create non-blurred images through the solar cell device, both of which are the critical challenges of the traditional perovskite solar cells that exploit a mesoscopic TiO_2_ scaffold [53]. For the thin film device structures of the perovskite solar cells, in general, the perovskite semiconductor layer is deposited onto charge-transporting layers on top of a transparent conductive oxide (TCO), such as indium tin oxide (ITO) and fluorine doped tin oxide (FTO). Then, the light-harvesting perovskite semiconductor materials with the electron or hole transporting layers, respectively, followed by the thick metallic films as a counter electrode, are deposited to complete the solar cell device. The simplest way to achieve the semitransparent characteristics would be to make a rear electrode optically transparent so that a certain portion of incident light can be transmitted after passing through the entire solar cell device. In this section, we discuss semitransparent perovskite solar cells utilizing a transparent electrode consisting of optically thin metallic film sandwiched or capped by wide band gap dielectric materials.

**Figure 1:**
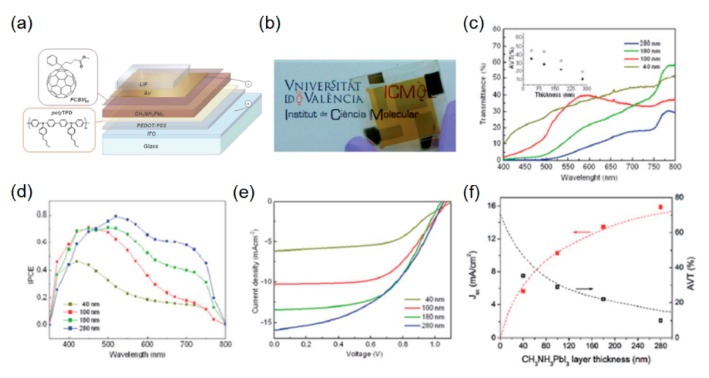
(**a**) Schematic layout of the semitransparent solar cell and chemical structures of the organic hole and electron blocking materials. (**b**) Photograph of the semitransparent solar cell having a 100 nm perovskite layer resulting in an average visible transmittance (AVT) of ~30% and a power conversion efficiency (PCE) of 6.4%. (**c**) Transmittance spectra through the complete device for different perovskite layer thicknesses. The inset shows the AVT values for the devices with (filled circle) and without (open circles) the semitransparent electrode. (**d**) Experimentally measured incident photon-to-current efficiency spectrum. (**e**) *J*-*V* characteristics of the best semitransparent devices comprising the Au/LiF electrode for different perovskite thicknesses. (**f**) Comparison between the experimentally obtained AVT (black open squares) and *J*_sc_ (red full squares) with the optical modeling (dashed lines). Reproduced with permission [13]. Copyright 2014, Royal Society of Chemistry.

Both aluminum (Al) and silver (Ag) have been widely used as electrode materials in many kinds of solar cell applications. However, easy oxidation properties of Al, which cause the thin metal film to be electrically non-conductive, and the very low stability of Ag, which formed Ag halide resulting in performance degradation over time, are the biggest concern, and both of these were unsuitable for the semitransparent perovskite solar cells [54]. By exploiting a proper buffer layer at the cathode, the reaction of Ag with the perovskite semiconductors can be prevented [55]. It has been demonstrated instead that an ultrathin gold (Au) film capped with a dielectric layer (lithium fluoride; LiF) with an optimized thickness that not only enhanced the transparency but retained high current density, all of which were modeled by the transfer matrix method, was utilized as the transparent electrode presented in Figure 1a [13]. Significantly minimized parasitic absorptions in the metal film were enabled by reducing the thickness of the metal layer and capping the ultrathin Au electrode with the LiF layer that could function as an anti-reflective (AR) coating. In addition to the AR effect, such a capping layer could protect the underlying Au electrode and simultaneously change the distribution of the electric field intensity in the solar cell device structure in order to improve the performance characteristics by optical interference behaviors. For hole and electron-transporting layers, highly transparent conducting polymers, poly(3,4-ethylenedioxythiophene):poly(styrenesulfonic acid) (PEDOT:PSS) and [6,6]-phenyl C_61_-butyric acid methyl ester (PCBM_60_) were employed, respectively. Such a device architecture implementation that removes metal oxides potentially paves the way toward the development of flexible semitransparent solar cells over a large area via roll-to-roll (R2R) process [56,57,58,59,60,61,62].

Figure 1b shows an optical image of solar cell with a 100 nm-thick perovskite layer (CH_3_NH_3_PbI_3_), exhibiting around 30% of the transmittance averaged over the entire visible wavelengths ranging from 400 to 800 nm with 6.4% of the PCE. As there is a trade-off between the PCE and the transmittance, increasing the thickness of the perovskite semiconductor film leads to stronger optical absorptions in the photoactive layer, thus generating the higher PCE. The solar cell device with the 180 nm-thick perovskite film displays the PCE of 7.3% with the lower average transmittance of 22% as compared to the 100 nm perovskite cell. As expected, the thickness of the perovskite layer is of critical importance to determine both the optical and electrical performances of the semitransparent perovskite solar cells. Figure 1c presents spectral transmission curves obtained through the complete solar cell devices with different active layer thicknesses, showing that enhanced transmission efficiency is attained by decreasing the photoactive layer thickness, as less light is absorbed by the perovskite film. As the perovskite film thickness decreases, it is apparent that the current density (*J*_sc_) also decreases, which is attributed to less absorptions in the photoactive layer and hence fewer generated photogenerated charges, as can be seen in Figure 1d–f. It is observed that the device with a thinner perovskite layer presents a notable decrease in incident photon-to-current efficiency (IPCE) at wavelengths beyond 550 nm, since the perovskite materials have a large optical absorption coefficient at shorter wavelengths due to its band gap, which can also be validated by the spectral curves of transmittance that show higher transmission efficiency at longer wavelengths, regardless of the photoactive layer thickness as depicted in Figure 1c. However, one concern of the above work is that LiF is highly reactive to oxygen, water vapor, and nitrogen, resulting in performance degradation.

**Figure 2:**
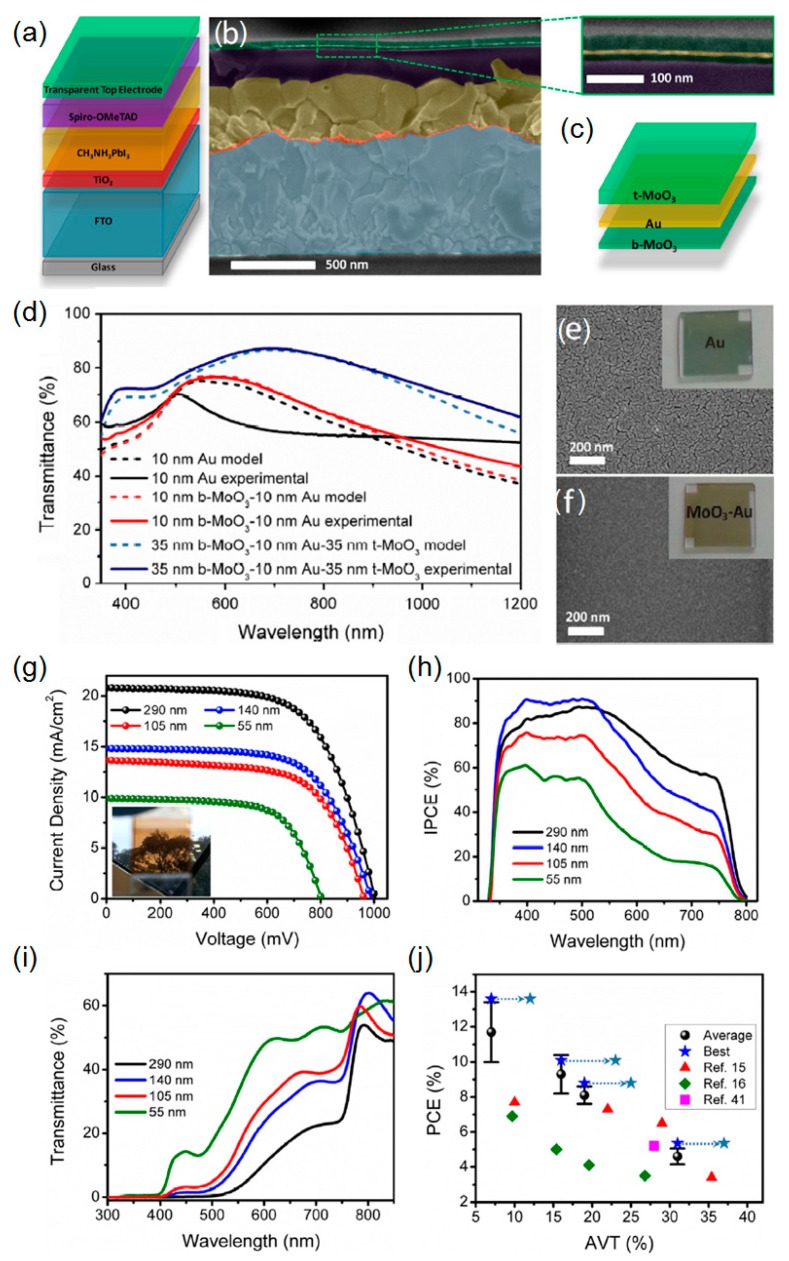
(**a**) Schematic illustration (not to scale) of the semitransparent perovskite solar cell architecture employing a dielectric-metal-dielectric (DMD) multilayer electrode atop. (**b**) SEM image in cross section of a complete device. (**c**) Enlarged view of the multilayer top electrode and schematic of its structure. (**d**) Simulations (shaded dashed lines) and experimental data (solid lines) showing the transmittance of Au (black), b-MoO_3_/Au (red) and b-MoO_3_/Au/t-MoO_3_ (blue). (**e**) SEM image of Au film. (**f**) SEM image of b-MoO_3_/Au film. The insets show photos of the two samples. (**g**) *J*-*V* curves of the semitransparent perovskite solar cells under air mass (AM) 1.5 (1 sun) illumination. All devices were illuminated from the fluorine-doped tin oxide (FTO) side. The inset shows a photograph of the fabricated device. (**h**) Incident photon-to-current efficiency (IPCE) spectra and (**i**) transmittance spectra of complete perovskite solar cells with different CH_3_NH_3_PbI_3_ film thicknesses. (**j**) Plot of the PCE (average and best) as a function of AVT (370–740 nm) and comparison with other semitransparent perovskite solar cells. The arrows show the performance of our best devices if the AVT is calculated between 400 and 800 nm, for a fair comparison with previously published results. Reproduced with permission [14]. Copyright 2015, Elsevier.

It has recently been demonstrated that the further improved PCE can be achieved by adopting a dielectric-metal-dielectric (DMD) multilayered rear electrode configuration [14,63]. When the two dielectric layers with proper thickness are separated by the thin metal film, a destructive interference at a certain wavelength in the visible range occurs to create anti-reflective coating effects, thereby improving the transmission efficiency, while the electrical conductivity is primarily determined by the thickness of the metal film. Such highly transparent conducting electrodes with various dielectric layers, including molybdenum trioxide (MoO_3_), tungsten trioxide (WO_3_), and vanadium pentoxide (V_2_O_5_), have been extensively investigated showing great promise in diverse optoelectronic devices, such as other types of the solar cells, light emitting diodes and photodetectors [1,18,64,65,66,67,68,69]. Recently, it has been demonstrated that adding a small amount of Al during the Ag deposition allows ultra-thin (e.g., 7 nm) and ultra-smooth Ag films to be formed with a long term stability [70,71,72]. Such semitransparent conductor films may be suitable for creating the semitransparent perovskite solar cells. However, as explained earlier, neither Ag nor Al are good candidate electrode materials for the semitransparent perovskite solar cell devices, in general. In this regard, Au could be a promising alternative for the rear electrode material, which can be sandwiched by any dielectric materials described above, since they are all conductive. However, when the thickness of the Au film is thinner than 10 nm, Au atoms accumulate by surface diffusion, which results in a rough surface [69]. Without the wetting layer underneath the thin Au film, typically, the surface shows a rough morphology with a grain size that is fairly large as compared to the wavelength of incident light. It has been found that the rough Au film on a glass appears greenish and bluish in color which arises from the presence of a localized surface plasmon resonance (LSPR), whereas an ideal thin Au film shows a yellowish color due to the interband transition of Au at 468 nm. Such non-smooth surfaces can induce light scattering loss and excite the LSPR, both of which lower the transparency, and also reduce sheet electrical resistance, thus suggesting that having the nucleation seeding layer underneath is essential to realizing a transparent electrode with high transparency and high conductivity. In particular, MoO_3_ has been largely employed in combination with the thin Au layer, as MoO_3_ film (even 1–2 nm thickness) can provide a good wettability of surface for Au material, thus leading to continuous, uniform, and ultrathin Au film. Gaspera *et al.* applied this strategy to the development of the semitransparent perovskite solar cells where the DMD rear electrode comprising MoO_3_-Au-MoO_3_ is utilized, as illustrated in Figure 2a. In this study, the continuous, homogeneous, and thin CH_3_NH_3_PbI_3_ active layer (~50 nm) is deposited by a gas-assisted spin casting method, thus enabling high efficiency and high visible transparency [73].

As seen from enlarged views of the scanning electron microscopy (SEM) image presented in Figure 2c,e,f, it is obvious that the ultrathin and smooth Au film is achieved when deposited on MoO_3_. It should also be noted that the thin Au film on the MoO_3_ layer provides much reduced sheet electrical resistance of ~13 Ω/sq as compared with the bare thin Au film on the glass that exhibits ~250 Ω/sq. The electrical conductivity can be significantly improved by increasing the thickness of the Au film. However, the transmission efficiency drops very rapidly, which is not a desired property for the transparent electrode. Figure 2d illustrates simulated and measured spectral curves of transmittance of the bare Au film, Au on the bottom MoO_3_ (b-MoO_3_) and Au sandwiched between the top (t-MoO_3_) and bottom MoO_3_ layers, presenting that the DMD electrode layout shows the highest transmission efficiency among them. It is important to note that the thickness of the b-MoO_3_ layer is of critical importance, particularly to determine the electrical performance of the perovskite solar cell, as its limited conductivity can affect the efficient extraction of the photogenerated charges from the hole transporting layer. From the optimization process, the optimal thickness range is found to be less than 20 nm. When the b-MoO_3_ is thicker than 20 nm and on top of the hole transporting layer, Spiro-OMeTAD, it has been shown that the PCE slightly decreases, which is mainly due to the reduction in fill factor (FF). This is attributed to the fact that it is difficult to completely extract the charges from the Spiro-OMeTAD layer with the thicker b-MoO_3_ film, which has also been observed previously with the MoO_3_/Al rear electrode for the perovskite solar cells [74]. As described above, the deposition of the perovskite active layer with high quality is enabled by the recently developed gas-assisted solution method. As the optical performance characteristics of the semitransparent perovskite solar cells are also influenced by the thickness of the active layer, several perovskite solar cell devices with different active layer thickness, ranging from ~50 to ~300 nm, are prepared by varying the precursor concentration. Figure 2g presents the *J*-*V* curves of the fabricated semitransparent solar cells with different active layer thickness, all of which are measured under air mass (AM) 1.5 illumination from the FTO side. The corresponding IPCE spectra are given in Figure 2h. As can be seen from these two figures, the thicker perovskite semiconductor layers absorb a more substantial amount of visible light, which leads to a large photocurrent generation. All the devices show relatively constant open-circuit voltage (*V*_oc_) and FF, except the device with ~50 nm thick perovskite active layer, which is attributed to the fact that pinholes, predominantly resulting from both the surface roughness of the FTO substrate and the thin perovskite film thickness. Figure 2i depicts the measured transmission spectra, presenting that the device with the thin perovskite film (~55 nm) exhibits the highest average visible transmittance (AVT) (averaged between 370 and 740 nm) of 31% (PCE = 5.3%), while achieving 19% (PCE = 8.8%), 16% (PCE = 10.1%), and 7% (PCE = 13.6%) of the AVT from ~105, ~140, and ~290 nm thick perovskite films, respectively. It is noticed that the appearance of the fabricated semitransparent perovskite solar cells changes from a neutral color to a brownish color as the thickness of the perovskite film increases. The plot that compares the device performance with what has recently been reported is given in Figure 2j. The highest PCE with the comparable AVT is attained by exploiting a highly efficient and highly transparent DMD multilayer electrode as the rear electrode of the semitransparent perovskite solar cells.

### 2.2. Solution-Processed Ag Nanowires

**Figure 3:**
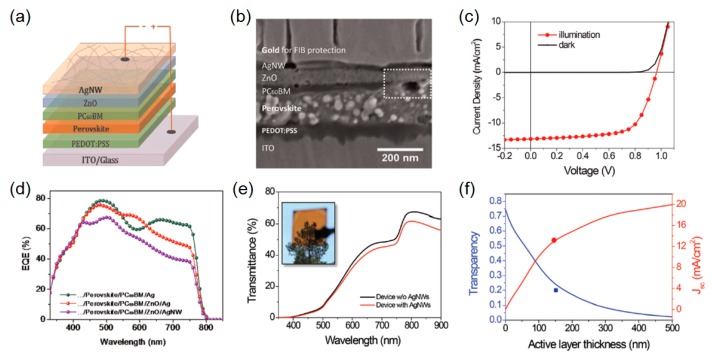
(**a**) Schematic structure of the devices with solution-processed top AgNWs. (**b**) Cross sectional SEM image of the whole device stack. The white dashed rectangle shows the area where perovskite is not covered by [6,6]-phenyl C_61_-butyric acid methyl ester (PCBM_60_). (**c**) *J*-*V* curves of the devices measured under illumination (red curve) and in the dark (dark curve). (**d**) External quantum efficiency (EQE) characteristics of the three types of devices studied in this work. (**e**) Transmittance spectra of the device without and with AgNW top electrodes, which were measured before and after AgNWs deposition. The inset shows the photo of a complete semitransparent device. (**f**) Calculated transparency and *J*_sc_ as a function of perovskite layer thickness. The blue and red dots are the experimental visible transparency (21.5%, corresponding to 28.4% AVT of the device) and *J*_sc_ values. Reproduced with permission [10]. Copyright 2015, Royal Society of Chemistry. PEDOT:PSS: poly(3,4-ethylenedioxythiophene):poly(styrenesulfonic acid); ITO: Indium Tin Oxide.

Although semitransparent perovskite solar cells can be achieved by employing thin metal films or multilayer electrodes, the high-vacuum evaporation method needs to be involved, which is not compatible with large-scale and low-cost applications. In particular, the Au is not an ideal material for the transparent electrode due to its high cost and low transparency in the near-infrared (NIR) spectral region, the latter of which notably hinders tandem solar cell construction. Recent works have demonstrated that solution-processed Ag nanowires (AgNWs) have shown great promise in achieving high transparency over a broad range of wavelengths with a low sheet resistance, thereby providing an opportunity for the extensive use of these solution-processed transparent electrodes for a multitude of other solar applications. Due to the poor compatibility of Ag with the perovskite semiconductor, however, exploring the Ag-based electrodes has remained largely unexploited in the perovskite solar cells. To address this challenge, Guo *et al.* introduced the zinc oxide (ZnO) nanoparticles-based thin interfacial layer between AgNWs and the electron transporting layer, which can efficiently prevent the diffusion of the Ag atoms into the perovskite material and also ensure ohmic contact at the top electrode. The device structure, which consists of PEDOT:PSS/CH_3_NH_3_PbI_3-x_Cl_x_/PCBM_60_/ZnO/AgNWs on the ITO/glass substrate, is schematically depicted in Figure 3a. Without inserting an additional ZnO electron transport layer between AgNWs and PCBM_60_, it is difficult for the charges to be efficiently extracted to the electrode, which is ascribed to a large energy barrier between PCBM_60_ and AgNWs. In addition, ZnO nanoparticles are highly compatible with evaporated Ag as well as solution-processed AgNWs, thus greatly serving not only as a capping layer but an ohmic contact formation at the ZnO-Ag interface for efficient electron extraction, both of which are responsible for achieving high values of *V*_oc_ and FF [75,76,77]. It should be noted that eliminating possible shunt-paths is enabled by completely and uniformly overlaying both the perovskite and PCBM_60_ layers beneath the top AgNW electrode with the ZnO interlayer. The optimal thickness of the ZnO layer is found to be between 60 and 90 nm. When the thickness of the ZnO layer is too thin, there is a possibility that Ag atom diffusion occurs through the thin ZnO layer, reducing the built-in potential and hence the *V*_oc_ of the solar cell devices. For the deposition of the AgNW electrode, a spray coating method is adopted as it can create a uniform thin film without damaging the underlying layer due to its very fast drying characteristic, which is difficult to achieve with both spin-coating and doctor-blading. In Figure 3b, a cross-sectional view of the fabricated semitransparent perovskite solar cell device structure is depicted, clearly showing that the interface between AgNWs and ZnO is fairly distinct. This implies that spray-coated AgNWs can be well supported by the underlying ZnO layer. The electrical performance characteristics, including *J*-*V* curve and external quantum efficiency (EQE) spectrum, of the semitransparent perovskite solar cell with the AgNW top electrode are given in Figure 3c,d, respectively. The semitransparent cell exhibits 13.18 mA cm^−2^ of *J*_sc_, which is relatively lower than the *J*_sc_ (15.72 mA cm^−2^) of a control sample that has a thick Ag electrode. This is attributed to the fact that the reflected light intensity from the semitransparent AgNW top electrode is low, whereas the opaque electrode of the control sample provides the strong reflections that can be harvested by the perovskite active layer. The PCE achieved from the semitransparent cell that has a ~150 nm thick perovskite active layer is 8.49%, along with 28.4% of AVT. As shown in Figure 3d, the control device exhibits 10% higher quantum efficiency in the visible wavelength range than the semitransparent cell with AgNWs, which corresponds well to the decrease in the *J*_sc_ due to the lower reflections. The measured spectral curves of transmittance of the devices before and after the AgNW deposition are depicted in Figure 3e, presenting that more than 50% of the transmission are over the wavelength range from 600 to 900 nm, thus opening important possibilities to further enhance the performance characteristics through the realization of the tandem solar cells. Figure 3f depicts the dependence of the thickness of the perovskite active layer on both the transparency and the *J*_sc_, all of which are estimated by the calculation using the transfer matrix method. As the active layer thickness increases, more significant visible light absorptions occur, consequently leading to higher *J*_sc_ values with lower transmission efficiency. The calculated results are in good agreement with the measured data when a perovskite active layer thickness of ~150 nm is used in the experiment. It is important to note that the semitransparent perovskite solar cells with the AgNW top electrode described in this study show greatly improved stability as compared to what has been observed so far. This is because having a compact ZnO interface layer can physically separate the perovskite light-absorbing medium from the AgNW top electrode, thereby hindering the Ag atoms from forming Ag halide by chemical reactions, which causes performance degradation. This finding provides the possibility of realizing the tandem solar cells by exploiting the semitransparent perovskite solar cells with a highly efficient and highly transparent AgNW top electrode as a top cell, which can be mechanically stacked or placed on top of the bottom cell with a tunnel junction [3,78].

### 2.3. Carbon Nanotube- and Graphene-Based Transparent Electrodes

**Figure 4:**
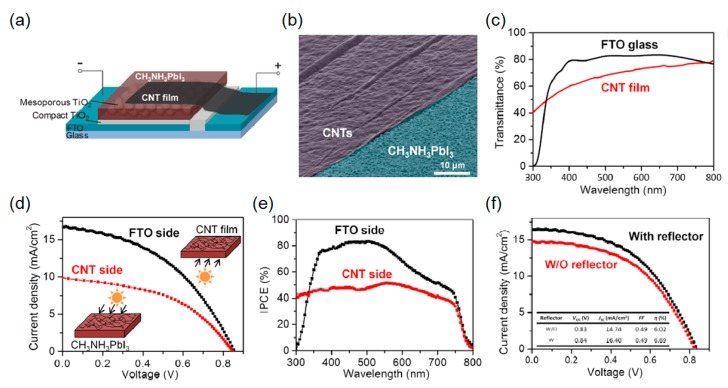
(**a**) Schematic diagram of CH_3_NH_3_PbI_3_ perovskite solar cell with carbon nanotube (CNT) film electrode. (**b**) Tilted SEM image of CH_3_NH_3_PbI_3_ perovskite substrate (blue) partly covered by CNT film (purple). (**c**) UV-vis transmittance of a CNT film and FTO glass. (**d**) Light *J*-*V* curves of CH_3_NH_3_PbI_3_ perovskite/CNTs solar cell with illumination from the FTO and from the CNT side, under conditions of AM1.5 100 mW cm^−2^. (**e**) IPCE of a CH_3_NH_3_PbI_3_ perovskite/CNTs solar cell with illumination from the FTO and from the CNT side. (**f**) Light *J*-*V* curves of a CH_3_NH_3_PbI_3_ perovskite/CNTs solar cell with and without an Al reflector. Reproduced with permission [12]. Copyright 2015, American Chemical Society.

Recent studies have revealed that carbon nanotubes (CNTs) have been utilized as electrodes due to their great chemical steadiness, high electrical conductivity, efficient charge collection, and low cost. In addition, other functionalities, including flexibility and high transparency, could be attained with the CNT electrodes [12,40,79,80,81,82]. Li *et al.* exploited the laminated CNT networks as the top electrode of the semitransparent perovskite solar cells [12]. In this study, the conventional perovskite solar cell device architecture based on a mesoporous TiO_2_ scaffold was used as presented in Figure 4a. A thin and dense TiO_2_ layer was deposited by spray pyrolysis on top of the FTO substrate, followed by the mesoporous nanocrystalline TiO_2_ layer. The light-harvesting perovskite semiconductor layer was deposited by employing a sequential deposition method [83]. Then, the freestanding thin CNT network film, which was synthesized using a floating catalyst chemical vapor deposition (CVD) technique, was placed on the perovskite active layer to complete the device structure [84]. The transfer process of the freestanding CNT film was performed in ambient atmosphere at room temperature. Note that the adhesion between the perovskite layer and the CNT film was improved by dropping a few droplets of toluene. This was due to the fact that the surface tension occurring during vaporization of the toluene pulled the CNT network film toward the active layer by van der Waals force without dissolving the active layer. This allowed the CNT network to be successfully transferred onto the perovskite layer in a conformal manner, which led to an enhanced electrical contact. In Figure 4b, a tilted SEM image shows the thin CNT film (purple) transferred on the perovskite active layer (blue), presenting that the active layer is conformally coated by the flexible CNT film with some wrinkles that are resulting from the manual transfer.

As the thickness of the CNT network film was very thin (20–50 nm), the optically transparent property was achieved and therefore the device could be illuminated from both the FTO and CNT sides. Figure 4c depicts the measured transmission spectrum of the bare FTO glass substrate and the CNT film, the latter of which shows relatively low transmission efficiency. The *J*-*V* characteristics of the fabricated semitransparent perovskite solar cells with the CNT top electrode under the standard AM1.5 solar spectrum from the FTO and CNT side are shown in Figure 4d. The PCE of the device with the FTO side illumination is 6.29% (*V*_oc_ = 0.86 V, *J*_sc_ = 16.7 mA cm^−2^, and FF = 44%), while the corresponding PCE is 3.88% (*V*_oc_ = 0.85 V, *J*_sc_ = 9.9 mA cm^−2^, and FF = 46%) when illuminated from the CNT side. This lower PCE with the CNT side illumination primarily arises from the small *J*_sc_ value, which is attributed to the slightly lower transmission of the CNT film, leading to the limited absorptions in the active layer. This similar trend is also observed in the IPCE spectrum as described in Figure 4e. Furthermore, the lower IPCE for CNT side illumination may be affected by the inferior charge collection efficiency of CNT network film. Therefore, Spiro-OMeTAD that is widely used in the existing perovskite solar cells as the hole transport layer can be incorporated into the thin CNT network film to provide better coverage over the surface of the perovskite layer as compared to the bare CNT network film. Meanwhile, the absorption edge of the perovskite semiconductor is around 800 nm, so the IPCE spectra with illumination on both sides rapidly drop around the cut-off wavelength. The IPCE spectrum when illuminated from the FTO side shows lower photocurrent generation at the wavelength below 350 nm, which is due to strong absorptions of both the FTO and TiO_2_. The *J*_sc_ value can also be improved by introducing the opaque metal layer at the backside of the device as exhibited in Figure 4f, presenting that the *J*_sc_ of the device increases to 16.4 mA cm^−2^. It should be noted that the PCE of the device with the CNT top electrode can be further improved by eliminating impurities on the CNT surface that leads to the low sheet resistance.

**Figure 5:**
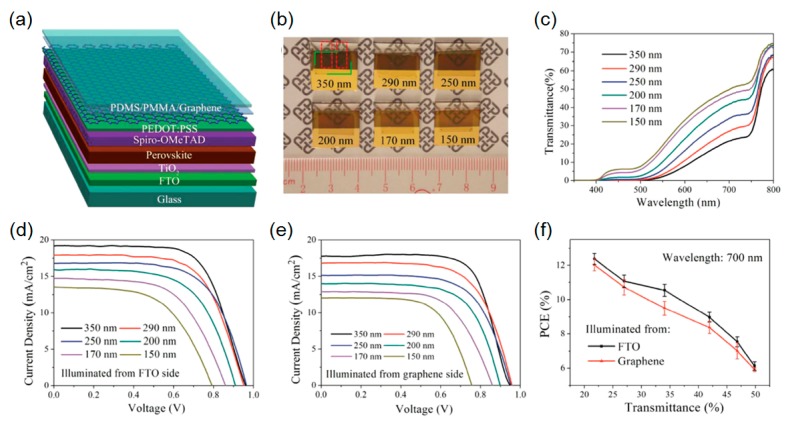
(**a**) Schematic view of a semitransparent perovskite solar cell employing a graphene based transparent top electrode. (**b**) Photos of semitransparent perovskite solar cells with transparent graphene electrodes. The thicknesses of the perovskite layers in the six devices are approximately 350, 290, 200, 170, and 150 nm, respectively. (**c**) Transmittance spectra of the above devices. *J*-*V* characteristics of the semitransparent solar cells illuminated from (**d**) the FTO side and (**e**) the graphene side. (**f**) The average PCEs of the semitransparent perovskite solar cells as a function of the transmittance at the wavelength of 700 nm. Reproduced with permission [35]. Copyright 2015, Wiley. PDMS: Polydimethylsiloxane; PMMA: Poly(methyl methacrylate); Spiro-OMeTAD: 2,2’,7,7’-tetrakis(*N*,*N*-di-*p*-methoxyphenylamine)-9,9-spirobifluorene.

It has been reported that a graphene material possesses a lot of attractive features as compared to ITO and FTO, such as high optical transparency, high electrical conductivity, mechanical robustness, chemical stability, and low cost, thus providing a new pathway to remarkably enhanced performance characteristics of various types of solar cells. With the help of its fascinating properties described above, the semitransparent perovskite solar cells have been demonstrated by laminating CVD-grown multilayer graphene as the top electrode as shown in Figure 5a. The device architecture comprises a thin and compact TiO_2_ layer (electron transport layer), perovskite light-absorbing semiconductor (photoactive layer), Spiro-OMeTAD/PEDOT:PSS (hole transport layers) and single-layer CVD graphene on top of the FTO glass substrate. A thin PEDOT:PSS layer is pre-deposited in order to improve the electrical conductivity of the graphene film, which is ascribed to the fact that the Fermi level of the PEDOT:PSS (5.0 eV) is higher than the Dirac point of graphene (4.6 eV), thus leading to an electrostatically-doped graphene layer for more hole injections [37]. As the PEDOT:PSS is highly transparent in the visible range, introducing the thin PEDOT:PSS layer does not have an influence on the transmission efficiency of the transparent electrode. In addition to the increased conductivity of the graphene film by the doping effect of PEDOT:PSS, it directly contacts with the Spiro-OMeTAD layer by van der Waals force, which also improves the adhesion of the graphene electrode to the perovskite active layer. Figure 5b illustrates photographs of the fabricated graphene-based semitransparent perovskite solar cell devices with different perovskite layer thicknesses from 150 to 350 nm. Note that the two-layer graphene films, which are prepared by layer-by-layer stacking process in order to further improve the electrical conductivity, are used in all semitransparent devices [85]. It is clear that the optical transparency becomes lower as the perovskite layer thickness increases, which can also be found in Figure 5c, presenting that the measured spectral curves of transmittance of the devices exhibit high transparency with decreasing thickness of the perovskite absorber. Figure 5d,e show the *J*-*V* curves of the devices having different perovskite absorber layer thicknesses with the FTO and graphene side illumination, respectively. The best optical and electrical performances of the semitransparent device are achieved with a 350 nm thick perovskite light-harvesting layer, showing a PCE of 12.37% with a transmittance of ~23% at 700 nm. Increasing the thickness of the perovskite absorber layer leads to the high photocurrent generation enabled by the strong optical absorptions in the active layer. It is also found that the *V*_oc_ value decreases from 0.95 to 0.76 V when the perovskite layer thickness is thinner than 200 nm, which would be attributed to deficient coverage of the perovskite film. Figure 5f depicts the relationship between the averaged PCE of the semitransparent perovskite solar cells employing the graphene film as the top electrode when illuminated from both the FTO and graphene sides and the optical transmittance at 700 nm. The transmission efficiency of ~50% at 700 nm while generating the PCE of ~6% is attained with the device having a 150 nm-thick perovskite light-absorbing semiconductor layer, which shows a relatively higher PCE value than the previous works having the similar average transparency. This would be enabled by the high transparency and high conductivity of the graphene film.

### 2.4. Transparent Conductive Oxides

**Figure 6:**
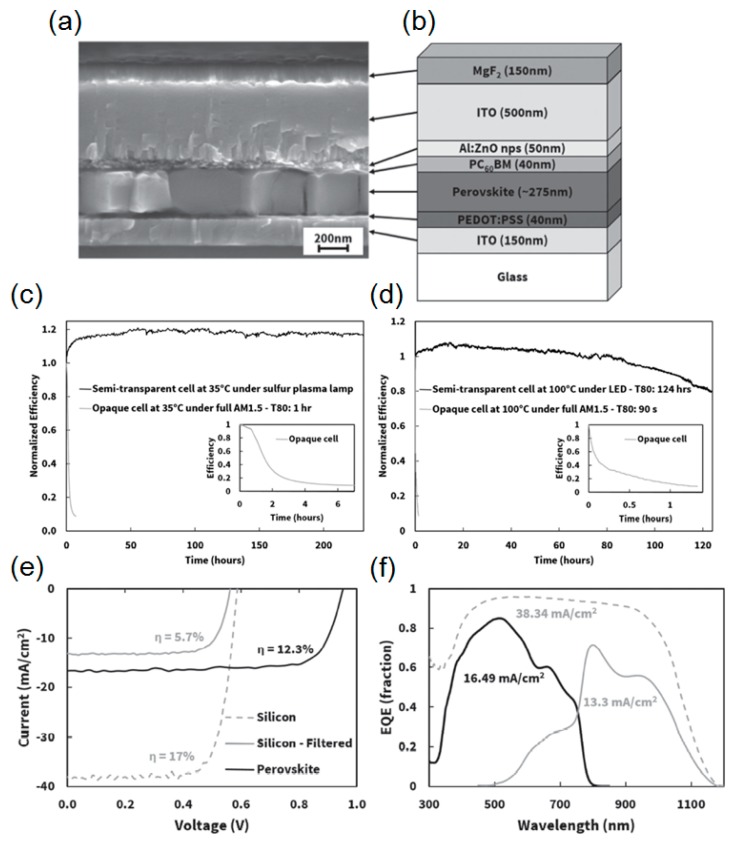
(**a**) A cross-sectional SEM image and (**b**) illustrative schematic of the semitransparent inverted perovskite device architecture showing the ITO electrode encapsulation layer. Thermal stability of ITO-capped perovskite solar cells at (**c**) 35 and (**d**) 100 °C compared to the opaque device with aluminum-doped zinc oxide (AZO) and Al/Ag. (**e**) *J*-*V* curves of the mechanically stacked perovskite/silicon (Si) tandem solar cell with the maximum power of the tandem calculated from the addition of the perovskite and Si cells. (**f**) External quantum efficiency spectra of original mono-Si, perovskite, and filtered Si solar cells. Reproduced with permission [48]. Copyright 2016, Wiley.

Although there have been a large number of attempts to develop a transparent electrode, as described above, both the optical and electrical performances of the conventional TCOs widely used in industry are still far superior to all other developed transparent electrodes. Many studies utilizing TCO layers such as ITO, hydrogenated indium oxide (In_2_O_3_:H), aluminum-doped zinc oxide (AZO) and indium zinc oxide (IZO) as the transparent top electrode have been performed. In particular, the ITO electrode has the lowest sheet resistance with the highest optical transparency among many TCOs. The biggest concern of exploiting such high-performance ITO electrodes in the perovskite solar cells, however, is that high temperature during the sputter deposition and post annealing should be involved, which can significantly degrade the device performance, since the perovskite light-absorbing materials are highly sensitive to processing conditions. It has been demonstrated that introducing a buffer layer, MoO_x_, between the perovskite absorber layer and the TCO electrode enables the underlying active layers to be protected from damage during the deposition of TCO [46,49]. However, a large extraction barrier could be created by a chemical reaction of the iodine in the perovskite semiconductors with the MoO_x_ layer, hence suggesting that the long-term stability remains a great challenge still to be addressed [86]. To resolve the aforementioned issues, Bush *et al.* exploited the solution-processed nanoparticles based on a wide band gap semiconductor, zinc oxide (ZnO), which could serve as not only an efficient electron transport layer but the buffer layer. This led to the deposition of the ITO electrode by the sputtering method without performance degradation. The device structure layout of the semitransparent perovskite solar cells with the ITO electrode and the corresponding cross-sectional view of the SEM image are given in Figure 6b,a, respectively. However, the efficient charge carrier extraction to the ITO electrode was hindered by the large barrier existing at the ZnO-ITO interface, which could be ascribed to the misaligned work functions of those two materials. The device could temporarily function well with increasing temperature while giving the lower performance back at room temperature. By employing aluminum-doped (2 mol%) zinc oxide (AZO) that was optimized for the device operation at 25 °C, the interfacial barrier was removed. Moreover, magnesium fluoride (MgF_2_) was deposited on top of the ITO electrode as the AR coating. The thermal stability of the semitransparent perovskite solar cells capped with the ITO electrode was explored by illuminating the both the semitransparent cell with ITO and the control cell with the opaque metal electrode (Al/Ag) at the maximum power point at 35 and 100 °C with no encapsulation. Figure 6c,d present the stability results obtained at 35 and 100 °C, respectively. As can be seen from inset images, it is apparent that the electrical performance of the control devices with the opaque Al/Ag at both 35 and 100 °C rapidly degrades over a short period of time (shorter than 2 h). This is likely because corrosion of the metal electrode occurs, which could be due to the HI from CH_3_NH_3_I. In contrast, the device employing the ITO capping layer resolves such difficulties simply because of the effective prevention of moisture and halide corrosion. The semitransparent cell tested at 35 °C shows almost steady efficiency for longer than 250 h and it takes 124 h to attain 80% of the efficiency of the semitransparent cell at 100 °C, thus validating the great thermal and ambient robustness of the ITO electrode. The tandem solar cell was experimentally demonstrated and the results are provided in Figure 6e,f. The semitransparent perovskite solar cells capped with the ITO electrode were placed on top of a monocrystalline Si solar cell, both of which were separately measured, thus forming a mechanically stacked tandem configuration. Eighteen percent (12.3% from the top perovskite cell; 5.7% from the bottom Si cell) of the PCE from the tandem solar cell was achieved.

### 2.5. Microstructured Arrays of Perovskite “Islands”

**Figure 7:**
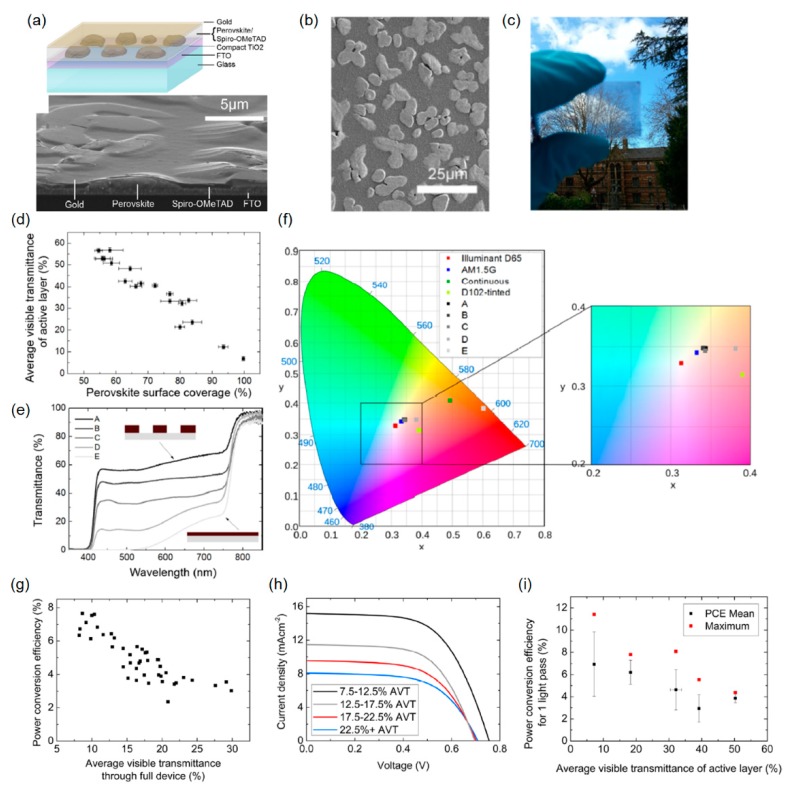
(**a**) Schematic diagram of semitransparent neutral-colored perovskite solar cells showing the architecture of the dewet planar perovskite heterojunction solar cell. Tilted cross-sectional SEM image of a full semitransparent solar cell showing the perovskite islands coated with Spiro-OMeTAD. (**b**) SEM image of the top surface of a representative film of perovskite islands (paler regions) on a TiO_2_-coated FTO substrate. (**c**) Photograph through a typical semitransparent perovskite film formed on glass, demonstrating neutral color and semitransparency. (**d**) Dependence of average visible transmittance of the active layer on perovskite surface coverage. (**e**) Transmittance spectra of active layers of a selection of dewet perovskite devices. Diagrammatical representations of the most and least transparent films are shown as insets. (**f**) Color coordinates of the films with transmittance spectra shown in (d) under AM1.5 illumination, on the CIE xy 1931 chromacity diagram, and the enlarged central region. Color coordinates of a thin continuous perovskite film, a D102 dye-tinted cell, the D65 standard daylight illuminant, and AM1.5 illumination are also shown. (**g**) Power conversion efficiencies for a batch of individual solar cells with ~10 nm Au electrodes, plotted as a function of full device AVT. (**h**) Average *J*-*V* characteristics for the cells plotted in (g). The curves are numerical averages of the *J*-*V* characteristics for individual cells split into the AVT intervals shown, with 5–15 cells per interval. (**i**) Power conversion efficiency plotted as a function of active layer AVT for a different batch of cells with thicker gold electrodes. The PCE plotted represents that which is attainable with an entirely transparent cathode (not a thick gold cathode). It has been corrected to remove current generated in the second pass. Each point represents the mean of at least 14 individual devices, and the maximum PCE for the champion device in each interval is plotted. In all cases, PCE was extracted from *J*-*V* measurements performed under 100 mW cm^−2^ AM1.5 illumination. Reproduced with permission [9]. Copyright 2014, American Chemical Society.

Instead of employing the transparent electrodes to create the semitransparent perovskite solar cells, a novel approach based on a microstructure architecture of the perovskite “islands” enabled by a morphological control was proposed by Eperon *et al.* The basic idea is that the device is not completely covered by the perovskite light-absorbing semiconductor in microscale with the thin Au layer, thereby producing semitransparency. The device has the thick perovskite active layer to fully absorb a broad range of the visible light, showing a black appearance, and simultaneously has the discontinuous areas (*i.e*., dewetted islands) to create optical transparency. This scheme can produce a neutral color that is different from what the works examined above achieved, all of which show reddish or brownish colors due to a relatively large optical absorption coefficient of the perovskite absorber materials at the shorter wavelengths. The planar heterojunction solar cell device layout comprising n-type dense TiO_2_, perovskite active layer, p-type Spiro-OMeTAD, and thin Au film on top of the FTO substrate is given in Figure 7a. As can be seen from the bottom image of Figure 7a displaying the tilted cross-sectional SEM image, the thin Spiro-OMeTAD layer infiltrates the gaps between the perovskite islands covering the microstructured architecture. Figure 7b describes the top SEM view of the dewetted regions of the perovskite absorbing film. An optical image clearly presenting that the device shows neutral-colored transparency is given in Figure 7c. In Figure 7d, a plot showing the relationship between the perovskite surface coverage and the visible transmittance averaged from 370 to 740 nm is illustrated. As the extent of the perovskite surface coverage increases, the AVT decreases due to the absence of the perovskite “islands”. Varying the amount of the dewetted regions, which is controllable by a processing temperature and a solvent selection, leads to different optical transparency. The transmission spectra of several samples, which are made by using different solvents and annealed at different temperatures, are provided in Figure 7e. It is clear that the majority of the fabricated devices show the transmittance featuring flat spectral characteristics, mostly over the entire visible wavelength, which ranges from 400 to 700 nm. The color-neutrality is estimated by calculating the color coordinate described on the CIE 1931 chromaticity diagram, as shown in Figure 7f. The product of the measured transmission spectrum by the standard AM1.5 solar spectrum is quantified. The estimated color coordinates of most devices are fairly close to both the AM1.5 spectrum and D65 reference, suggesting that the great neutral color is achieved. Figure 7g,h present the PCE as a function of AVT and the *J*-*V* characteristics of the fabricated devices, respectively. Approximately 8% (AVT ~7%) and ~3.5% (AVT ~30%) of the PCE are achieved. In general, the *J*_sc_ increases as the amount of dewetting decreases (*i.e*., better perovskite surface coverage), which leads to lower AVT. Figure 7i presents the relationship between the PCE estimated only for one light passage through the device and the AVT. The portion of the photocurrent generated by the reflection from the metal electrode surface is removed, thereby implying that the achievable PCE with a completely transparent electrode instead of the non-optimum thin Au layer. From this investigation, it is demonstrated that the PCE of over 8% with ~30% of the AVT could be attained, which is analogous to the commercially-available semitransparent solar cells. It should be noted that the multi-color generation is also enabled by incorporating chemical pigment or color dyes into the layers where incident light goes through. The performance characteristics of the semitransparent perovskite solar cells can be further improved by employing high-performance top and bottom electrodes such as TCOs and graphenes, engineering the band gap of the perovskite absorber layers, or applying the AR coatings.

The performance characteristics of the developed semitransparent perovskite solar cells are summarized in Table 1.

## 3. Multi Colored Perovskite Solar Cells

### 3.1. Band Gap Tuning by a Chemical Management

**Figure 8:**
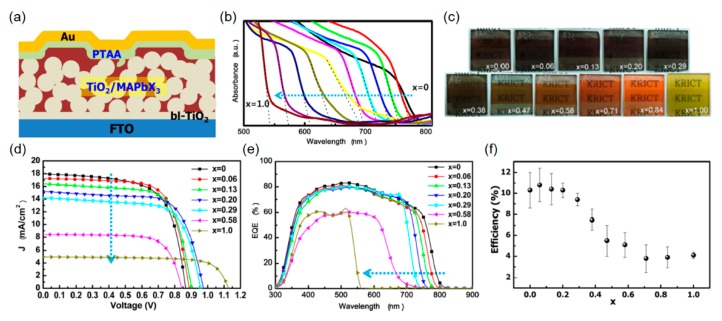
(**a**) Device configuration of the hybrid heterojunction solar cell consisting of the 3D TiO_2_/ Methyl Ammonium (MA)PbX_3_ bilayer nanocomposites, polytriarylamine (PTAA) layer, and Au layer on the blocking TiO_2_ layer (bl-TiO_2_) coated FTO substrate. (**b**) UV−vis absorption spectra of FTO/bl-TiO_2_/mp-TiO_2_/MAPb(I_1−x_Br_x_)_3_/Au cells measured using an integral sphere. (**c**) Photographs of 3D TiO_2_/MAPb(I_1−x_Br_x_)_3_ bilayer nanocomposites on FTO glass substrates. (**d**) *J−V* characteristics of the heterojunction solar cells based on MAPb(I_1−x_Br_x_)_3_ (x = 0, 0.06, 0.13, 0.20, 0.29, 0.58, 1.0). (**e**) Corresponding external quantum efficiency (EQE) spectra. (**f**) Power conversion efficiencies of the heterojunction solar cells as a function of Br composition (x). Reproduced with permission [87]. Copyright 2013, American Chemical Society.

In this section, we will summarize the recent studies that focus on the development of colored and semitransparent perovskite solar cells. It has been reported that the band gap of CH_3_NH_3_PbX_3_ (CH_3_NH_3_: Methyl Ammonium (MA); X = I, Br or Cl) perovskite semiconductor can be tuned by changing halide ions. This is ascribed to the fact that electronic energies are greatly dependent upon the effective exciton mass. The difference between an ionic radius of I¯ (2.2 Å) and Br¯ (1.96 Å) leads to different perovskite structure; MAPbBr_3_ exhibits a cubic perovskite structure, whereas MAPbI_3_ shows a contorted 3D perovskite structure. As the energy level of the conduction band of the mesoscopic TiO_2_ scaffold is lower than that of both MAPbBr_3_ and MAPbI_3_, the electrons can be efficiently transported to the mesoporous TiO_2_ layer upon light illumination. Schematic view of the heterojunction perovskite solar cell structure is given in Figure 8a. Figure 8b presents the measured UV-visible absorption profiles of mesoporous TiO_2_/MAPb(I_1−x_Br_x_)_3_ (0 ≤ x ≤ 1), which is controlled by the alloying of MAPbI_3_ and MAPbBr_3_. It is obvious that both the band gap of the perovskite absorber layer (e.g., 786 nm of band edge with x = 0; 544 nm of band edge with x = 1) and the corresponding appearances (e.g., dark brown with x = 0; yellow with x = 1) are easily tuned via the chemical compositional control as shown in Figure 8b,c. A band edge of the absorption spectrum shifts toward the shorter wavelengths with increasing Br content. *J*-*V* characteristics, EQE spectrum, and the corresponding PCE of the fabricated perovskite solar cells with different Br content are provided in Figure 8d–f. As the amount of Br in the alloying mixture increases (*i.e*., large x), the band gap of the perovskite light-harvesting layer increases, leading to higher *V*_oc_ and lower *J*_sc_ values, which can be clearly seen from both the measured *J*-*V* curves and the EQE spectra shown in Figure 8d,e.

### 3.2. Microcavity Integrated Cathodes

**Figure 9:**
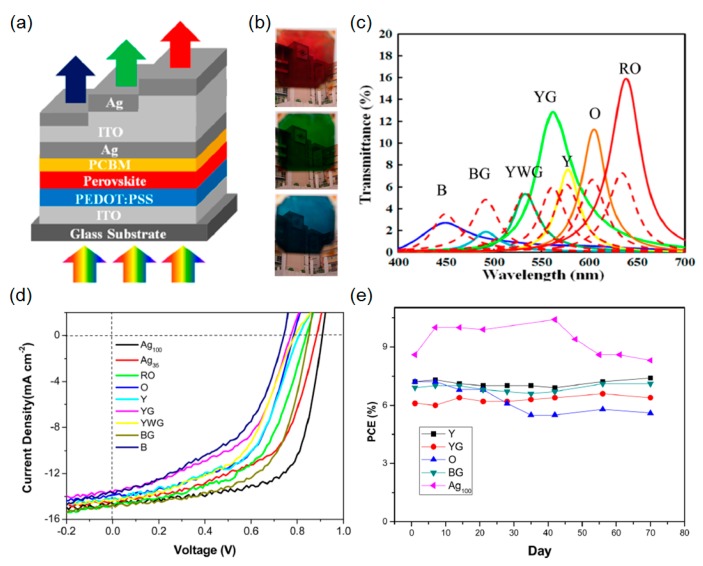
(**a**) Schematic representation of microcavity-based semitransparent perovskite solar cells. (**b**) Images of fabricated colored solar cell devices. The background image can be clearly seen through our fabricated samples with various colors. (**c**) Simulated (dashed lines) and measured (solid lines) spectral transmittance curves of the perovskite solar cell devices. (B: blue; BG: blue-green; YWG: yellowish-green; YG: yellow-green; Y: yellow; O: orange; RO; reddish-orange) (**d**) *J*-*V* characteristics of various colored devices under illumination with AM 1.5G solar simulated light (100 mW cm^-2^). (Ag_100_:Ag = 100 nm; Ag_35_:Ag = 35 nm) (**e**) Long-term performance of the perovskite cells. Reproduced with permission [17]. Copyright 2016, American Chemical Society.

Although several colors are created by the compositional control of the perovskite materials, the color generation ranges only from brown to yellow. In addition to the limited color tunability, both the *J*_sc_ and the *V*_oc_ will be greatly affected by the band gap tuning, thus leading to the lower PCE with the large content of Br. To address these challenges, it has been demonstrated that the microcavity in a metal-dielectric-metal configuration is integrated with the perovskite solar cells at the top electrode for achieving the optical transparency as presented in Figure 9a [17]. Incident light passes through the entire device structure, which has a relatively thin perovskite active layer, and the constructive interference occurs at a certain wavelength (called a resonance) in the microcavity-integrated electrode to produce the desired colors, which can be readily tuned by varying the thickness of the dielectric layer. Increasing the thickness of the optical space dielectric film allows the resonance (*i.e*., transmission peak) to appear at longer wavelengths, thereby achieving red color generation. The calculated (dashed lines) and measured (solid lines) of the spectral transmittances and the distinctive red, green, and blue colored perovskite solar cells are illustrated in Figure 9c,b, respectively. It is apparent that the transmission efficiency of the red colored cell is relatively higher than that of the blue colored cell, which arises from the strong absorption of the ~150 nm-thick perovskite absorbing layer at the shorter wavelength region. Experimentally measured *J*-*V* curves of the fabricated semitransparent cells with various colors are shown in Figure 9d. Over 7% of the PCE with ~8% average transmittance is achieved from most devices. The chemical and ambient steadiness of the fabricated semitransparent colored perovskite solar cells is investigated in a glovebox, and the corresponding long-term performance results are given in Figure 9e. As examined in the previous section discussing the ITO incorporated semitransparent cells, the improved long-term stability property could be attributed to the strong resistance of the ITO capping layer with respect to the moisture and halide corrosion.

## 4. Conclusions

During the past several years, the perovskite solar cell has been highlighted as a strong candidate for the next generation of solar cell technology due to its superior properties, which can produce comparable performance to the conventional Si-based solar cell, even with low cost solution-based processes. For that purpose, significant efforts have been made to improve their performance, and their PCE has finally reached over 20%. However, to commercialize this technology, many issues, such as the toxicity of materials commonly utilized to produce high performances, the long-term stability, and the applicability to large area fabrication process without loss of performance should be resolved. Therefore, if we can extend the application of this technology to multi-functional off-grid power sources, different from the conventional utilization in rooftops and power plants, we can find another opportunity to commercialize this technology for our daily life, even without achieving high PCE comparable to Si solar cell technology. In this context, the efforts to produce neutral or multi-colors from the perovskite solar cell, introduced in this article, can be alternative approaches to commercialize this technology. For this purpose, designing various advanced device architectures, which can not only maximize the photocharge generation with minimum usage of light in the device (e.g., light trapping, low reflection, low parasitic absorption in the non-photocharge generating layers, utilization of UV and IR parts, *etc*.) but also manipulate the desired colors, can be possible ways, and synthesizing new perovskite materials having high quantum efficiency would be also helpful. By incorporating color tunable multi-functionality to perovskite solar cells, it is expected to be one of the most promising candidates in future solar cell technology.

## Figures and Tables

**Table 1:** Performance metrics of the semitransparent perovskite solar cells.

Electrodes	*J*_sc_ (mA cm^−2^)	*V*_oc_ (V)	FF (%)	PCE (%)	AVT (%)	Ref.
Thin Au	10.30	1.07	57.90	6.41	29 (400–800 nm)	[13]
Thin Ag	15.87	1.00	69.90	11.04	20.8 (380–750 nm)	[60]
MoO_x_/Au-Ag/MoO_x_	14.60	1.05	75.10	11.50	N/A	[63]
MoO_x_/Au/MoO_x_	14.70	0.95	65.00	10.10	16 (400–800 nm)	[14]
AgNWs	13.18	0.96	66.80	8.49	28.4 (350–800 nm)	[10]
AgNWs	15.87	0.96	69.68	10.55	25.5 (380–750 nm)	[77]
AgNWs	17.50	1.03	71.00	12.70	77 at 800 nm	[3]
CNT	18.10	1.00	55.00	9.90	N/A	[12]
Graphene	19.17	0.96	67.22	12.37	22.5 at 700 nm	[35]
ITO	16.50	0.95	77.00	12.30	N/A	[48]
ITO	14.50	0.82	51.90	6.20	55 (800–1200 nm)	[49]
In_2_O_3_:H	17.40	1.10	73.60	14.20	72 (800–1150 nm)	[50]
Microstructure	12.00	0.85	63.00	6.40	12.5 (370–740 nm)	[9]
ZnO:Al	16.70	1.03	70.3	12.1	71 (800–1000 nm)	[46]
